# Knowledge and Awareness of Volume Assessment Among Emergency and Intensive Care Physicians in Saudi Arabia

**DOI:** 10.7759/cureus.82913

**Published:** 2025-04-24

**Authors:** Assad Bafarag, Abdullah A Balkhair, Ahmed S Qanat, Safinaz Alshiakh, Sadeen Ashour

**Affiliations:** 1 Intensive Care Unit, King Abdulaziz University Hospital, Jeddah, SAU; 2 Emergency Medicine, King Abdulaziz University Hospital, Jeddah, SAU; 3 Medicine, King Abdulaziz University, Jeddah, SAU

**Keywords:** ed, em, emergency department, emergency medicine, icu, intensive care unit, knowledge, saudi arabia, volume

## Abstract

Background: Fluid volume measurement in the emergency department and intensive care unit (ICU) is critical for patient care. This study aimed to assess the knowledge of volume assessment among emergency medicine (EM) and ICU physicians in Saudi Arabia.

Materials and methods: A cross-sectional study was conducted among EM and ICU physicians using an online questionnaire. Data were collected on participants’ demographics, work-related information, confidence in volume assessment, use of point-of-care ultrasonography, and knowledge and practices of volume assessment.

Results: Of the 114 physicians surveyed, 92 (80.7%) were aged 25-35 years, 65 (57%) were male, 70 (61.4%) were EM physicians, and 68 (59.6%) had fewer than five years of practice. ICU physicians demonstrated significantly higher knowledge that, in mechanically ventilated patients, a distensibility index of >18% indicates fluid responsiveness. In contrast, EM physicians had a higher proportion of correct responses regarding the indications for using Swan-Ganz catheters. The most commonly used method for volume assessment was physical examination (83, 72.8%), and the most frequently used laboratory biomarker was serum lactate (65, 57%). The majority (85, 74.6%) used focused cardiac assessments, including evaluation of the inferior vena cava, for volume assessment. ICU physicians reported significantly higher use of Doppler ultrasound for volume assessment. Only 17 (14.9%) physicians demonstrated a good level of knowledge of volume assessment, with no significant associations found between knowledge level and participants’ demographics, work experience, or confidence in volume assessment.

Conclusions: A poor understanding of fluid volume assessment was observed among EM and ICU physicians in Saudi Arabia. Training on the principles of volume assessment is needed.

## Introduction

Measuring fluid volume in emergency departments and intensive care units (ICUs) is critical for patient care. Accurate determination and regulation of fluid balance can significantly impact patient prognosis, either promoting recovery or leading to deterioration [1].

Volume evaluation is a vital component of the therapeutic decision-making process in intensive care. Differentiating between hypovolemia, euvolemia, and hypervolemia is crucial, as each state has a significant impact on patient outcomes. Inadequate volume assessment can lead to severe consequences, including untreated shock, unnecessary fluid administration, pulmonary edema, impaired organ function, and increased mortality [2].

Determining a patient's volume status requires a multifaceted approach that incorporates clinical assessments, hemodynamic measurements, and the interpretation of diagnostic imaging and laboratory tests. Traditional methods rely on physical indicators such as skin turgor, capillary refill time, and vital signs. More advanced techniques include measuring central venous pressure, using pulmonary artery catheters, and employing ultrasound to evaluate metrics such as inferior vena cava (IVC) diameter and respiratory fluctuations [3]. While Saudi medical personnel receive training in assessing fluid volume, there is still room for improvement in their knowledge and methods. Studies highlight the need for expanded educational initiatives focused on effective hydration therapy and advanced assessment methodologies [4,5]. With the shift toward evidence-based medicine, continuous professional development aligned with current scientific advancements is essential.

Improving practices can involve standardizing fluid volume assessment protocols across healthcare institutions, integrating advanced technologies and methods into routine care, conducting hands-on workshops and simulation training, emphasizing fluid management in ongoing medical education, and promoting research on fluid resuscitation and management. Non-invasive ultrasound techniques, particularly IVC collapsibility studies, are gaining popularity due to their accuracy and minimal patient discomfort. Additionally, complex hemodynamic measurements such as stroke volume variation are gaining traction for their ability to provide precise insights into a patient’s volume status [6].

However, several barriers complicate the accurate assessment of volume status, including educational gaps, resistance to adopting new technologies, inconsistent standards, and clinical inertia. The shift toward non-invasive methods, such as point-of-care ultrasonography (POCUS), may encounter resistance due to limited awareness and promotion among healthcare professionals [7].

Appropriate fluid management is essential to prevent serious complications or fatal outcomes. Overhydration can lead to pulmonary edema and heart failure, whereas under-resuscitation may result in poor tissue perfusion, multiple organ failure, and shock [8]. Emergency departments and ICUs care for patients with complex medical conditions, requiring a thorough understanding of fluid volume status [4]. For example, patients with septic shock require carefully tailored fluid management to avoid fluid overload.

Saudi Arabia demonstrates a strong commitment to medical education and ongoing professional development through its training programs, fellowships, and specialized seminars, which address topics such as fluid management and hemodynamic monitoring [5]. Expanded POCUS training enables clinicians to perform non-invasive volume assessments at the bedside. Enhancing the skills of Saudi emergency medicine (EM) and ICU physicians in fluid volume assessment is crucial for delivering high-quality intensive care. To improve patient outcomes, it is essential to strengthen educational and training programs, standardize clinical procedures, and incorporate advanced hemodynamic monitoring technologies. Educational efforts should prioritize practical applications of volume assessment techniques and encourage active engagement with various monitoring tools [9].

Periodic reviews of clinical practices, research into local procedures, and international collaboration can help address knowledge gaps and promote quality improvement [10]. Considering Saudi Arabia's unique patient demographics and healthcare challenges, emphasis should be placed on region-specific assessment strategies, cultural competence, and ethically grounded clinical decision-making. As emergency and intensive care medicine continue to evolve, physicians must pursue ongoing professional development to maintain proficiency in core competencies, including volume assessment, which is crucial for improving patient outcomes in emergency departments and ICUs in Saudi Arabia [9,11,12].

Modern technologies and monitoring systems are being increasingly adopted in emergency departments and ICUs in Saudi Arabia. Significant progress has been made in the healthcare sector, aligning with the objectives of Saudi Vision 2030 to improve healthcare quality. Nevertheless, further improvement is needed in the fluid volume assessment practices of EM and ICU physicians [13].

This study aimed to assess the current knowledge and application of fluid volume assessment methods among Saudi EM and ICU physicians, highlighting the urgent need for improvement in this critical area.

## Materials and methods

Study design, setting, and time

This quantitative cross-sectional study was conducted in Saudi Arabia from January 2024 to January 2025. Following an extensive literature review, the questionnaire was validated by a panel of four experts, including two EM consultants with ultrasound fellowship training and two senior ICU consultants, to ensure clinical relevance and content validity. Before implementing the survey, a pilot study was conducted with a small group of EM and ICU physicians.

Study participants

The inclusion criteria included all physicians working in emergency departments and ICUs, while the exclusion criteria were physicians not working in these two settings. A purposive sample was selected.

Data collection

Data were collected through an online questionnaire distributed via Google Forms (Google LLC, Mountain View, CA, USA). Participation was voluntary, and informed consent was obtained electronically. All data were anonymized to ensure participant confidentiality and privacy.

The first section of the questionnaire assessed participants’ demographic characteristics, work-related information (specialty, postgraduate level, and years of practicing in EM/ICU), and their level of confidence in volume assessment methods and the use of POCUS. The second section evaluated physicians’ knowledge and awareness of volume assessment, while the third section focused on their related practices.

A score of "1" was awarded for each correct answer, and a score of "0" was given for each incorrect or "I don't know" response, with a maximum total of 21 points. If a participant’s score was below 60% of the total, their overall knowledge level was considered poor; a score of 60% or higher was rated as good knowledge [14,15].

Ethical considerations

Ethical approval for the study was obtained from the Research Ethics Committee of King Abdulaziz University (approval number: 147-24; approval date: March 12, 2024).

Data analysis

The data were analyzed using SPSS Statistics version 26 (IBM Corp., Released 2019. IBM SPSS Statistics for Windows, Version 26.0. Armonk, NY: IBM Corp.). To examine the relationship between the variables, the chi-squared test (χ²) was used for qualitative data presented as numbers and percentages. The mean and standard deviation (mean ± SD) were used for quantitative variables. A p-value of less than 0.05 was considered statistically significant.

## Results

Of the 114 physicians studied, 92 (80.7%) were between the ages of 25 and 35. There were 65 males (57%), and 96 (84.2%) were of Saudi nationality. Among them, 70 (61.4%) were EM physicians, and 44 (38.6%) were ICU physicians. Regarding postgraduate level, 50 (43.9%) were resident "board," and 34 (29.8%) were general practitioners (GPs). More than half (68, 59.6%) had less than five years of practice. Approximately 46 (40.4%) reported confidence in the volume assessment method, while 44 (38.6%) were confident in the use of POCUS (Table 1).

**Table 1:** Distribution of studied participants by demographic characteristics, work-related data, and level of confidence regarding the volume assessment method and the use of POCUS (N=114) EM: emergency medicine, ICU: intensive care unit, GP: general physician, POCUS: point-of-care ultrasonography

Variable	n (%)
Age (years)	
25-35	92 (80.7)
36-45	15 (13.2)
46-55	6 (5.3)
>55	1 (0.9)
Gender	
Female	49 (43)
Male	65 (57)
Nationality	
Saudi	96 (84.2)
Non-Saudi	18 (15.8)
Specialty	
EM physician	70 (61.4)
ICU physician	44 (38.6)
Postgraduate level	
GP	34 (29.8)
Resident "board"	50 (43.9)
Senior registrar/registrar "board certified physician"	14 (12.3)
Fellow	7 (6.1)
Consultant	9 (7.9)
How many years have you been practicing EM\ICU?	
<5 years	68 (59.6)
5-15 years	26 (22.8)
16-25 years	17 (14.9)
>25 years	3 (2.6)
What’s your Level of confidence regarding the volume assessment method?	
Confident	46 (40.4)
Neutral	54 (47.4)
Unconfident	14 (12.3)
What’s your level of confidence regarding the use of POCUS?	
Confident	44 (38.6)
Neutral	53 (46.5)
Unconfident	17 (14.9)

The participants' knowledge of volume assessment is illustrated in Tables 2-4. Forty-eight participants (42.1%) knew that orthostatic hypotension is a physical exam finding indicating low volume status. More than half (62, 54.4%) knew that 8-12 mmHg indicates normal central venous pressure. In comparison, 54 participants (47.4%) knew that three or more B-lines in a longitudinal plane between two ribs in two or more regions, bilaterally, indicate hypervolemia on a lung scan. About 61 participants (53.5%) knew that an IVC diameter ≤2.1 cm and collapsibility >50% during inspiration indicate fluid responsiveness in an IVC scan of a spontaneously breathing patient. In comparison, 51 participants (44.7%) knew that an IVC diameter >2.1 cm with collapsibility <50% during inspiration indicates hypervolemia on an IVC scan in a spontaneously breathing patient. Fifty-one participants (44.7%) knew that, in mechanically ventilated patients, a distensibility index of >18% indicates fluid responsiveness using the formula (maximum diameter - minimum diameter) / minimum diameter. As for the formula to calculate stroke volume variation, 53 participants (46.5%) correctly identified that it is SVV = (maximum SV - minimum SV) / mean SV.

**Table 2:** Comparison of EM and ICU physicians' knowledge and awareness of volume assessment (N=114) * correct answer SVV: stroke volume variation, SV: stroke volume, IVC: inferior vena cava, EM: emergency medicine, ICU: intensive care unit

Variable	Total	Specialty		χ2	p-value
	n (%)	EM physicians, n (%)	ICU physicians, n (%)		
Knowledge					
Which of the following physical exam findings indicates low volume status?					
Moist mucous membranes	16 (14)	11 (15.7)	5 (11.4)	1.78	0.619
Orthostatic hypotension*	48 (42.1)	30 (42.9)	18 (40.9)		
Urine output of 0.5-1 ml/kg/hr	16 (14)	11 (15.7)	5 (11.4)		
Increased skin turgor	34 (29.8)	18 (25.7)	16 (36.4)		
Which of the following indicates normal central venous pressure?					
8-12 mmHg*	62 (54.4)	33 (47.1)	29 (65.9)	4.39	0.222
12-16 mmHg	27 (23.7)	18 (25.7)	9 (20.5)		
16-20 mmHg	18 (15.8)	14 (20)	4 (9.1)		
20–24 mmHg	7 (6.1)	5 (7.1)	2 (4.5)		
N/A (not trained enough)	26 (22.8)	12 (17.1)	14 (31.8)	0.33	0.069
Which of the following indicates hypervolemia in a lung scan?					
Two or more A-lines in a longitudinal plane between two ribs in two or more regions bilaterally	20 (17.5)	13 (18.6)	7 (15.9)	3.11	0.374
Three or more A-lines in a longitudinal plane between two ribs in two or more regions bilaterally	24 (21.1)	17 (24.3)	7 (15.9)		
Two or more B-lines in a longitudinal plane between two ribs in two or more regions bilaterally	16 (14)	7 (10)	9 (20.5)		
Three or more B-lines in a longitudinal plane between two ribs in two or more regions bilaterally*	54 (47.4)	33 (47.1)	21 (47.7)		
What indicates fluid responsiveness in an IVC scan in a spontaneously breathing patient?					
IVC diameter ≤2.1cm and collapsibility >50% during inspiration*	61 (53.5)	34 (48.6)	27 (61.4)	3.6	0.307
IVC diameter >2.1 cm with collapsibility <50% during inspiration	25 (21.9)	15 (21.4)	10 (22.7)		
IVC diameter ≤3.1 cm with collapsibility >50% during inspiration	23 (20.2)	18 (25.7)	5 (11.4)		
IVC diameter >3.1 cm with collapsibility <50% during inspiration	5 (4.4)	3 (4.3)	2 (4.5)		
What indicates hypervolemia in an IVC scan in a spontaneously breathing patient?					
IVC diameter ≤2.1cm and collapsibility >50% during inspiration	16 (14)	12 (17.1)	4 (9.1)	4.93	0.177
IVC diameter >2.1 cm with collapsibility <50% during inspiration*	51 (44.7)	27 (38.6)	24 (54.5)		
IVC diameter ≤3.1 cm with collapsibility >50% during inspiration	12 (10.5)	6 (8.6)	6 (13.6)		
IVC diameter >3.1 cm with collapsibility <50% during inspiration	35 (30.7)	25 (35.7)	10 (22.7)		
In mechanically ventilated patients, which of the following is correct?					
Collapsibility of >50% during inspiration indicated hypovolemia	33 (28.9)	24 (34.3)	9 (20.5)	8.08	0.044
Collapsibility of <50% during inspiration indicated volume overload	20 (17.5)	15 (21.4)	5 (11.4)		
Distensibility index of >18% indicates fluid responsiveness using: (maximum diameter - minimum diameter)/minimum diameter)*	51 (44.7)	24 (34.3)	27 (61.4)		
Distensibility index of <18% indicated fluid responsiveness using: (maximum diameter - minimum diameter)/minimum diameter)	10 (8.8)	7 (10)	3 (6.8)		
Which of the following is the correct formula to calculate stroke volume variant?					
SVV (%) = (maximum SV- minimum SV) / mean SV*	53 (46.5)	35 (50)	18 (40.9)	2.63	0.452
SVV (%) = (maximum SV- minimum SV) / maximum SV	31 (27.2)	18 (25.7)	13 (29.5)		
SVV (%) = (maximum SV- minimum SV) / minimum SV	24 (21.1)	15 (21.4)	9 (20.5)		
SVV (%) = (maximum SV- minimum SV)	6 (5.3)	2 (2.9)	4 (9.1)		
Which of the following measures left ventricular preload?					
Systemic vascular resistance	17 (14.9)	14 (20)	3 (6.8)	5.62	0.131
Pulmonary capillary wedge pressure*	48 (42.1)	31 (44.3)	17 (38.6)		
Central venous pressure	17 (14.9)	9 (12.9)	8 (12.2)		
Left ventricular stroke volume index	42 (28.1)	16 (22.9)	16 (36.4)		
Which is the best measurement of contractility?					
Central venous pressure	17 (14.9)	9 (12.9)	8 (12.2)	2.07	0.722
Left ventricle stroke volume index*	37 (32.5)	26 (37.1)	11 (25)		
Cardiac output	42 (36.8)	25 (35.7)	17 (38.6)		
Pulmonary artery wedge pressure	7 (6.1)	4 (5.7)	3 (6.8)		
Right ventricle stress volume index	11 (9.6)	6 (8.6)	5 (11.4)		
Which factors may contribute to an underestimation of the cardiac output?					
Pulmonary hypertension	27 (23.7)	16 (22.9)	11 (25)	2.1	0.551
Aortic regurgitation*	34 (29.8)	18 (25.7)	16 (36.4)		
Right-to-left intracardiac shunt	36 (31.6)	24 (34.3)	12 (27.3)		
Tricuspid regurgitation	17 (14.9)	12 (17.1)	5 (11.4)		
In regards to PiCCOpuls system (Pulsion Medical Systems), it can record all of the following except*					
Stroke volume	24 (21.1)	15 (21.4)	9 (20.5)	1.7	0.635
Stroke volume variation	24 (21.1)	17 (24.3)	7 (15.9)		
Central venous pressure	42 (36.8)	23 (32.9)	19 (43.2)		
Pulse pressure variation*	24 (21.1)	15 (21.4)	9 (20.5)		
The PiCCO monitor combines pulse contour analysis and trans pulmonary thermodilution to provide a continuous measurement of					
Cardiac output	28 (24.6)	16 (22.9)	12 (27.3)	1.6	0.658
Cardiac output and intermittent assessment of extravascular lung water	35 (30.7)	22 (31.4)	13 (29.5)		
Cardiac output and intermittent assessment of intrathoracic blood volume and extravascular lung water*	37 (32.5)	25 (35.7)	12 (27.3)		
Cardiac output and intermittent assessment of intrathoracic blood volume	14 (12.3)	7 (10)	7 (15.9)		
Concerning checking for fluid responsiveness, select the incorrect option of the following					
Clinical assessment is an optimal tool to check fluid responsiveness*	33 (28.7)	16 (22.9)	17 (38.6)	6.29	0.178
Central venous pressure and pulmonary artery occlusion pressure predict fluid responsiveness poorly, though trend analysis may be useful	39 (34.2)	26 (37.1)	13 (29.5)		
Variation in arterial pulse pressure can predict fluid responsiveness in ventilated patients	20 (17.5)	16 (22.9)	4 (9.1)		
Arterial pulse pressure variation cannot be used to assess fluid response in spontaneously breathing patients	12 (10.5)	7 (10)	5 (11.4)		
Passive leg raising may aid in the prediction of fluid response in spontaneously breathing patients	10 (8.8)	5 (7.1)	5 (11.4)		

**Table 3:** Continuation of the comparison between EM and ICU physicians based on their knowledge and awareness of volume assessment (N=114) * correct answer IVC: inferior vena cava, SvO2: mixed venous saturation, EM: emergency medicine, ICU: intensive care unit

Variable	Total	Specialty		χ2	p-value
	n (%)	EM physicians, n (%)	ICU physicians, n (%)		
Central venous pressure of 4 mmHg is most likely associated with fluid responsiveness.					
True*	73 (64)	48 (68.6)	25 (56.8)	1.62	0.302
False	41 (36)	22 (31.4)	19 (43.2)		
SvO2 of 80% indicates hypoperfusion and a poor prognosis.					
True	64 (56.1)	35 (50)	29 (65.9)	2.77	0.096
False*	50 (43.9)	35 (50)	15 (34.1)		
In mechanically ventilated patients, the collapsibility index is used to assess the IVC using ultrasound.					
True	56 (49.1)	31 (44.3)	25 (56.8)	1.69	0.193
False*	58 (50.9)	39 (35.7)	19 (43.2)		
In the end, the expiratory occlusion test showed that holding our breath for 15 seconds increased cardiac output by more than 5%. This indicates fluid responsiveness.					
True*	69 (60.5)	42 (60)	27 (61.4)	0.02	0.885
False	45 (9.5)	28 (40)	17 (38.6)		
In the albumin challenge test, cardiac output will increase by more than 10% in a fluid-responsive patient.					
True*	73 (64)	48 (68.6)	25 (56.8)	1.62	0.203
False	41 (36)	22 (31.4)	19 (43.2)		
Swan-Ganz catheter indications include right-sided endocarditis, tricuspid and pulmonary mechanical valve prostheses, and arrhythmias.					
True	52 (45.6)	26 (37.1)	26 (59.1)	5.24	0.022
False*	62 (54.4)	44 (62.9)	18 (40.9)		

**Table 4:** Comparison between EM and ICU physicians regarding the best indicators for when a fluid bolus would be beneficial in a given case scenario, and the incorrect option related to fluid responsiveness for guiding fluid administration and hemodynamic monitoring (N=114) * correct answer EM: emergency medicine, ICU: intensive care unit

Variable	Total	Specialty		χ2	p-value
	n (%)	EM physician, n (%)	ICU physicians, n (%)		
A 68-year-old patient is admitted to the ICU post-emergency laparotomy for colonic perforation. On mechanical ventilation, with pressure support mode. He is becoming progressively more tachycardic and hypotensive despite receiving 3 L of Ringer's lactate. Regarding the previous case, which of the following options would be the best indicators that a fluid bolus would be of benefit?					
	Stroke volume variation of 14%	30 (26.3)	17 (24.3)	13 (29.5)	5.56	0.234
Central venous pressure 6 mmHg	24 (21.1)	18 (25.7)	6 (13.6)		
Passive leg raise increment in stroke volume of 12%*	41 (36)	22 (31.4)	19 (43.2)		
Pulmonary artery occlusion pressure of 10 mmHg	11 (9.6)	9 (12.9)	2 (4.5)		
Left ventricle end diastolic diameter of 4 cm in the parasternal long axis view	8 (7)	4 (5.7)	4 (9.1)		
Concerning fluid responsiveness to guide fluid administration and hemodynamic monitoring, which is the incorrect option?					
The goals of volume management are to optimize intravascular volume, cardiac output, tissue perfusion, and oxygen delivery to tissues. Insufficient volume administration may perpetuate hypoperfusion, and volume overload may result in organ congestion and dysfunction, either of which may increase morbidity and mortality	32 (28.1)	18 (25.7)	14 (31.8)	2.06	0.558
In non-intubated patients, the passive leg raising test and the mini-fluid challenge are not suitable*	53 (46.5)	31 (44.3)	22 (50)		
Extravascular lung water and pulmonary vascular permeability measured by trans pulmonary thermodilution directly reflect the risk of fluid leakage toward the interstitium and alveoli	19 (16.7)	14 (20)	5 (11.4)		
The presence of edema does not exclude the need for fluids	10 (8.8)	7 (10)	3 (6.8)		

Of the participants, 48 (42.1%) knew that pulmonary capillary wedge pressure measures left ventricular preload, and 37 (32.5%) knew that left ventricular stroke volume index is the best measurement of contractility. Only 34 (29.8%) knew that aortic regurgitation is a factor that may contribute to an underestimation of cardiac output. In comparison, only 21.1% knew that the PiCCOpuls system (Pulsion Medical Systems, Bavaria, Germany) cannot record pulse pressure variation. Almost one-third of the studied physicians (37, 32.5%) knew that the PiCCO monitor combines pulse contour analysis and transpulmonary thermodilution to provide continuous measurement of cardiac output and intermittent assessment of intrathoracic blood volume and extravascular lung water. Only 33 (28.7%) knew that clinical assessment could not check for fluid responsiveness. When EM physicians were compared to ICU physicians based on their responses to knowledge items, it was found that ICU physicians had a significantly higher prevalence of knowledge that in mechanically ventilated patients, a distensibility index of >18% indicates fluid responsiveness using the formula (maximum diameter - minimum diameter) / minimum diameter (61.4% vs. 34.3%) (p<0.05). On the other hand, no significant difference was found between EM physicians and ICU physicians for all other knowledge items (p>0.05) (Table 2).

Table 3 shows that most of the studied physicians (73, 64%) agreed that a central venous pressure of 4 mmHg is most likely associated with fluid responsiveness. Additionally, 69 (60.5%) agreed that in the end-expiratory occlusion test, if expiration is held for 15 seconds and cardiac output increases by more than 5%, this indicates fluid responsiveness. At the same time, 73 (64%) knew that in the albumin challenge test, cardiac output increases by more than 10% in a fluid-responsive patient. Fifty (43.9%) reported that it is false that a mixed venous saturation (SvO2) of 56% (80%) indicates hypoperfusion and a poor prognosis. In comparison, 58 (50.9%) reported that it is incorrect to use the collapsibility index to assess the IVC in mechanically ventilated patients on ultrasound. More than half (62, 54.4%) reported that it is false that Swan-Ganz catheter indications include right-sided endocarditis, tricuspid and pulmonary mechanical valve prostheses, and arrhythmias. It was revealed that EM physicians had a significantly higher percentage of those who correctly disagreed that Swan-Ganz catheter indications include right-sided endocarditis, tricuspid and pulmonary mechanical valve prostheses, and arrhythmias (p<0.05).

Only 41 (36%) participants knew that a 12% increase in stroke volume from a passive leg raise would be the best indicator that a fluid bolus would be beneficial. In comparison, 53 (46.5%) knew that, concerning fluid responsiveness to guide fluid administration and hemodynamic monitoring, the statement “in non-intubated patients, the passive leg raise test and the mini-fluid challenge are not suitable” is incorrect. No significant difference was found between the EM Physicians and the ICU Physicians for the previous two knowledge items (p>0.05) (Table 4).

Regarding the physicians’ practices related to volume assessment, the most commonly used methods were physical examination (83, 72.8%), POCUS (71, 62.3%), chest X-ray (59, 51.8%), and laboratory biomarkers (58, 50.9%). In comparison, the most commonly used lab biomarkers to predict a patient's volume status were serum lactate (65, 57%) and serum blood urea nitrogen (58, 50.9%). The majority (85, 74.6%) used focused cardiac assessment, including IVC assessment, to evaluate the patient’s volume. It was found that ICU physicians had a significantly higher prevalence of using Doppler ultrasound (e.g., assessing common carotid artery blood flow with passive leg raise) to evaluate a patient’s volume compared to EM physicians (36.4% vs. 17.1%) (p<0.05). On the other hand, no significant difference was found between the EM physicians and the ICU physicians in all other practices related to volume assessment (p>0.05) (Table 5).

**Table 5:** Comparison of EM and ICU physicians' practices of volume assessment (N=114) EVLW: extravascular lung water, EM: emergency medicine, ICU: intensive care unit, IVC: inferior vena cava, CO2: carbon dioxide

Variable	Total	Specialty		χ2	p-value
	n (%)	EM physicians, n (%)	ICU physicians, n (%)		
Practice					
What do you mostly use to assess volume status in your practice?					
Physical examination	83 (72.8)	54 (77.1)	29 (65.9)	1.72	0.189
Laboratory biomarkers	58 (50.9)	31 (44.3)	27 (61.4)	3.15	0.076
Chest X-ray	59 (51.8)	38 (54.3)	21 (47.7)	0.46	0.495
POCUS	71 (62.3)	42 (60)	29 (65.9)	0.4	0.526
Central venous pressure measurement	38 (33.3)	20 (28.6)	18 (40.9)	1.85	0.174
Pulmonary artery catheter pressure	22 (19.3)	14 (20)	8 (18.2)	0.05	0.811
What lab biomarker do you use to predict a patient's volume status?					
Serum sodium and osmolality changes	40 (35.1)	21 (30)	19 (43.2)	2.06	0.151
CO2 gap	42 (36.8)	21 (30)	21 (47.7)	3.64	0.059
Serum lactate	65 (57)	42 (60)	23 (52.3)	0.65	0.417
Serum uric acid	30 (26.3)	20 (28.6)	10 (22.7)	0.47	0.49
Serum blood urea nitrogen	58 (50.9)	35 (50)	23 (52.3)	0.05	0.813
Urine studies (urine specific gravity)	33 (28.9)	19 (27.1)	14 (31.8)	0.28	0.592
Natriuretic peptides	38 (33.3)	20 (28.6)	18 (40.9)	1.85	0.174
What method do you know for assessing a patient’s volume using ultrasound?					
EVLW	36 (31.6)	20 (28.6)	16 (36.4)	0.75	0.384
Focused cardiac assessment, including IVC assessment	85 (74.6)	56 (80)	29 (65.9)	2.82	0.093
Internal jugular vein assessment	40 (35.1)	23 (32.9)	17 (38.6)	0.39	0.529
Doppler ultrasound (e.g., common carotid artery blood flow with passive leg raise)	28 (24.6)	12 (17.1)	16 (36.4)	5.38	0.02

The mean knowledge score was 9.11 ± 3.09. Based on the knowledge score classification, only 17 (14.9%) of the studied physicians had a good knowledge level of volume assessment, while the majority (97, 85.1%) had a poor knowledge level.

Table 6 demonstrates that, although the prevalence of a good knowledge level of volume assessment was higher among younger physicians (25-35 years, 82.4%), males (76.5%), and those of non-Saudi nationality (82.4%), none of these associations were statistically significant (p>0.05). Additionally, the prevalence of good knowledge was higher among EM physicians (58.8%), residents (58.8%), those with less than five years of practice (76.5%), and those with neutral confidence in the volume assessment method and the use of POCUS. However, none of these associations were significant (p>0.05).

**Table 6:** Relationship between knowledge level of volume assessment among the studied physicians and their demographics, work-related data, confidence level regarding volume assessment methods, and the use of POCUS (N=114) POCUS: point-of-care ultrasonography

Variable	Knowledge level		χ2	p-value
	Poor knowledge, n (%)	Good knowledge, n (%)		
Age (years)				
25-35	78 (80.4)	14 (82.4)	1.54	0.673
36-45	12 (12.4)	3 (17.6)		
46-55	6 (6.2)	0 (0.0)		
>55	1 (1)	0 (0.0)		
Gender				
Female	45 (46.4)	4 (23.5)	2.08	0.079
Male	52 (53.6)	13 (76.5)		
Nationality				
Saudi	15 (15.5)	3 (17.6)	0.05	0.82
Non-Saudi	82 (84.5)	14 (82.4)		
Specialty				
EM physician	60 (61.9)	10 (58.8)	0.05	0.813
ICU physician	37 (38.1)	7 (41.2)		
Post-graduate level				
GP	31 (32)	3 (17.6)	2.12	0.713
Resident "board"	40 (41.2)	10 (58.8)		
Senior registrar/registrar "board certified physician"	12 (12.4)	2 (11.8)		
Fellow	6 (6.2)	1 (5.9)		
Consultant	8 (8.2)	1 (5.9)		
How many years have you been practicing EM\ICU?				
<5 years	55 (56.7)	13 (76.5)	2.79	0.424
5-15 years	23 (23.7)	3 (17.6)		
16-25 years	16 (16.5)	1 (5.9)		
>25 years	3 (3.1)	0 (0.0)		
What’s your level of confidence regarding the volume assessment method?				
Confident	41 (42.3)	5 (29.4)	1.19	0.551
Neutral	45 (46.4)	9 (52.9)		
Unconfident	11 (11.3)	3 (17.6)		
What’s your level of confidence regarding the use of POCUS?				
Confident	39 (40.2)	5 (29.4)	1.22	0.543
Neutral	43 (44.3)	10 (58.8)		
Unconfident	15 (15.5)	2 (11.8)		

**Figure 1:**
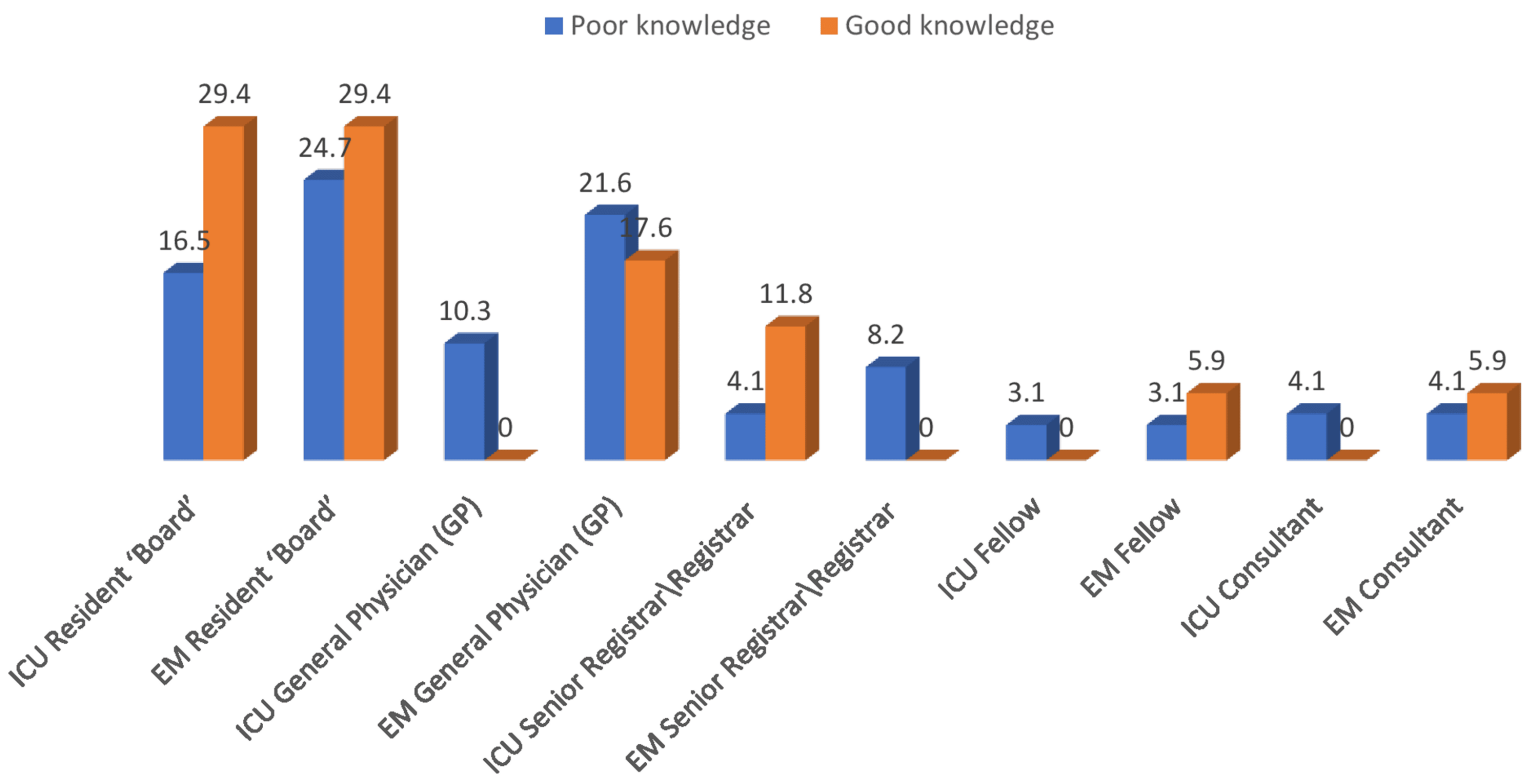
Relationship between knowledge levels of volume assessment and post-graduate levels across specialties N.B.: (χ2 = 7.95, p-value = 0.539) ICU: intensive care unit, EM: emergency medicine

When participants were categorized by postgraduate level in each specialty, no significant difference was found between categories in knowledge of volume assessment (p>0.05) (Figure 1).

## Discussion

This study aimed to evaluate the existing understanding and use of fluid volume measurement methods among EM and ICU physicians. The study's findings provided vital insights into the current state of knowledge, confidence, and practical application of fluid volume assessment procedures among EM and ICU physicians in Saudi Arabia.

A particularly concerning finding is that many respondents lack confidence in accurately determining volume status. Specifically, only 40.4% were confident in their assessment procedures, while 38.6% felt competent in using POCUS for this purpose. This sobering information emphasizes the urgent need for targeted educational activities to improve healthcare personnel's skills in fluid management procedures, which are critical for patient care.

Several studies have thoroughly established the vital importance of accurate volume assessment when treating critically ill patients. Misjudgments about fluid status, whether hypovolemia or hypervolemia, can have disastrous consequences, including increased morbidity and mortality [1,2,16]. The significant gaps in knowledge and self-assurance among the participating physicians highlight the urgent need for standardized training programs to improve clinicians' skills in this critical area of practice.

While it is promising that many physicians recognize the importance of POCUS in volume assessment, its implementation in routine clinical practice remains woefully inadequate. Existing literature supports this argument, revealing that, despite strong data demonstrating POCUS as a reliable and non-invasive method for assessing intravascular volume status, its widespread use is hindered by several constraints. These limitations include restricted access to necessary ultrasound equipment, insufficient training opportunities, and a lack of strong institutional support [17-19]. Nonetheless, evidence strongly suggests that POCUS can significantly improve diagnostic accuracy and clinical decision-making in critically ill patients, making it an essential tool in emergency departments and ICUs [20]. Overcoming the current reluctance to utilize this technology requires the development of structured training programs and seminars that can be seamlessly integrated into residency training and ongoing professional education.

The study found considerable regional variations in POCUS training and knowledge among physicians. Those working in urban and resource-rich areas were significantly more proficient in using ultrasonography than their counterparts in rural or under-resourced settings. According to reports, most non-academic and rural emergency departments in Saudi Arabia have poor work environments [21]. This trend indicates ongoing inequitable access to new medical technologies and training, ultimately leading to disparities in clinical practice standards among healthcare facilities [22]. Addressing these inequities is not merely a matter of fairness; it is essential to advancing healthcare quality. Healthcare policymakers must prioritize programs that ensure equitable access to training resources, essential equipment, and standardized techniques for measuring fluid volume throughout Saudi Arabia.

The current study found that only 53.5% of participants understood that an IVC diameter ≤2.1 cm with collapsibility >50% during inspiration suggests fluid responsiveness in spontaneously breathing patients. Meanwhile, 44.7% recognized that an IVC width >2.1 cm with collapsibility <50% on inspiration suggests hypervolemia in these patients. Although ultrasonographic evaluation is known to be dependent on the sonographer's experience, Fields et al. found that M-mode IVC diameter measurements performed by EM residents after a brief training course demonstrated a high degree of inter-rater reliability [23].

According to the current study's findings, only 14.9% of participants demonstrated a strong knowledge of volume evaluation. Aalam et al. reported that while all EM residency training programs in Saudi Arabia are of reasonably high quality, as measured by the Postgraduate Hospital Educational Environment Measure instrument, Saudi EM trainees felt less competent in managing multiple emergencies and perceived faculty supervision and educational interest as weaker [24]. These findings were attributed to fewer formal didactic sessions and limited simulation experience in Saudi Arabia. Aalam et al. recommended several improvements, including incorporating simulation into the EM curriculum and making relevant policy and regulatory adjustments.

The relationship between physician experience and confidence in volume assessment approaches is both important and informative. Physicians with more than 15 years of clinical practice were significantly more confident than their less experienced counterparts. These data highlight the critical role that clinical exposure plays in skill development, emphasizing the importance of rigorous hands-on training and mentorship programs that aim to bridge knowledge gaps among junior practitioners [25]. Furthermore, introducing simulation-based training into medical education provides a highly effective way for physicians to fine-tune their volume assessment skills in a safe and controlled setting before applying them in real-world scenarios [26].

Fluid management errors can result in longer hospital stays, increased healthcare costs, and higher morbidity and mortality rates among critically ill patients [27]. The ability to perform accurate and rapid volume assessments becomes crucial in emergencies such as septic shock, acute renal injury, and heart failure [28]. Consequently, incorporating systematic, evidence-based fluid management strategies is not only beneficial but also essential for improving patient outcomes and optimizing resource utilization.

Careful monitoring of volume status and fluid delivery is a key predictor of outcomes in critically ill patients [29]. Enhancing emergency care is therefore a vital step toward achieving universal health coverage, and the World Health Organization recognizes it as "an essential part of integrated healthcare delivery" [30].

A notable strength of this study is that it is the first conducted in Saudi Arabia to assess the level of knowledge of volume assessment among EM and ICU physicians. However, the use of a purposive sample necessitates future national studies with larger samples to validate the observed results.

## Conclusions

This study highlighted the poor knowledge of fluid volume assessment among EM and ICU physicians in Saudi Arabia, with the majority lacking essential proficiency. Structured training in both basic principles and advanced tools, such as POCUS, is crucial. Integrating comprehensive education, hands-on practice, and institutional support will enhance competency. Further research should assess the effectiveness of training and explore strategies to improve adoption. Encouraging continuous learning will help ensure better patient outcomes. Future national studies with larger sample sizes are recommended to confirm the findings of the current study.
